# Unveiling a Rare Case: Madras Motor Neuron Disease in an 18-Year-Old Patient

**DOI:** 10.7759/cureus.59812

**Published:** 2024-05-07

**Authors:** Keyur Saboo, Sourya Acharya, Sunil Kumar, Rajesh Sarode, Rinkle Gemnani

**Affiliations:** 1 Department of Medicine, Jawaharlal Nehru Medical College, Datta Meghe Institute of Higher Education and Research, Wardha, IND

**Keywords:** case report, sensory neural hearing loss, upper motor neuron disease, lower motor neuron disease, madras motor neuron disease

## Abstract

Madras motor neuron disease (MMND) is a rare childhood or juvenile motor neuron disease. Herein, we present a unique case of MMND in an 18-year-old patient, which challenges the conventional understanding of the disease's onset and progression. The patient, a previously healthy adolescent, presented with insidious onset and gradually progressive weakness of all four limbs, wasting, tongue fasciculation, and bilateral sensorineural hearing loss. Neurological examination revealed signs consistent with lower motor neuron involvement. Electromyography (EMG) and nerve conduction studies (NCS) supported the diagnosis of MMND. The patient's clinical course exhibited rapid deterioration, leading to significant functional impairment within a short timeframe. Treatment modalities, including supportive care and symptomatic management, were implemented; however, disease progression remained relentless. This case highlights the significance of considering MMND in the differential diagnosis of motor neuron diseases, even in young individuals. It highlights the importance of conducting more studies to comprehend the underlying mechanisms and consider potential therapeutic strategies for this uncommon ailment.

## Introduction

Madras motor neuron disease (MMND) is a rare neurodegenerative disorder characterized by progressive degeneration of motor neurons. This leads to muscle weakness, atrophy, and eventual paralysis. It was first identified in 1970 in Southern India and was described by Meenakshisundaram et al. [1]. Further reports of this case were reported from the south Indian city of Bangalore and other Indian towns [2]. MMND represents a distinct subtype of motor neuron disease (MND), with a prevalence primarily reported in South Indian populations. The disease follows a slow onset and gradual progression, leading to significant disability and reduced life expectancy. It affects males and females equally. This rare neuromuscular disease typically begins young and has a relatively mild course. Despite being recognized for 50 years, the exact cause remains unknown, but genetic factors, particularly autosomal recessive inheritance, are considered significant. Due to the small number of reported cases, it is classified as an orphan disease.

To date, only a limited number of MMND cases have been reported worldwide, predominantly from India and neighboring regions. Moreover, the occurrence of MMND in younger individuals, particularly adolescents, is exceedingly rare, with very few documented cases in the literature. Here, we present a detailed case report of MMND in an 18-year-old patient, highlighting the clinical features, diagnostic evaluation, disease progression, and therapeutic challenges encountered in managing this unique presentation We hope that this case will add to the depth of knowledge already available on motor neuron disorders and highlight the significance of taking this uncommon entity into account when making a differential diagnosis, particularly in younger patients. In addition, we stress the necessity for additional investigation to understand the pathophysiological mechanisms causing MMND and investigate promising targeted therapy strategies to enhance patient outcomes. To assess several causative factors, including genetic, environmental, and some ethnic factors, a multifaceted approach is needed [2].

## Case presentation

**Figure 1:**
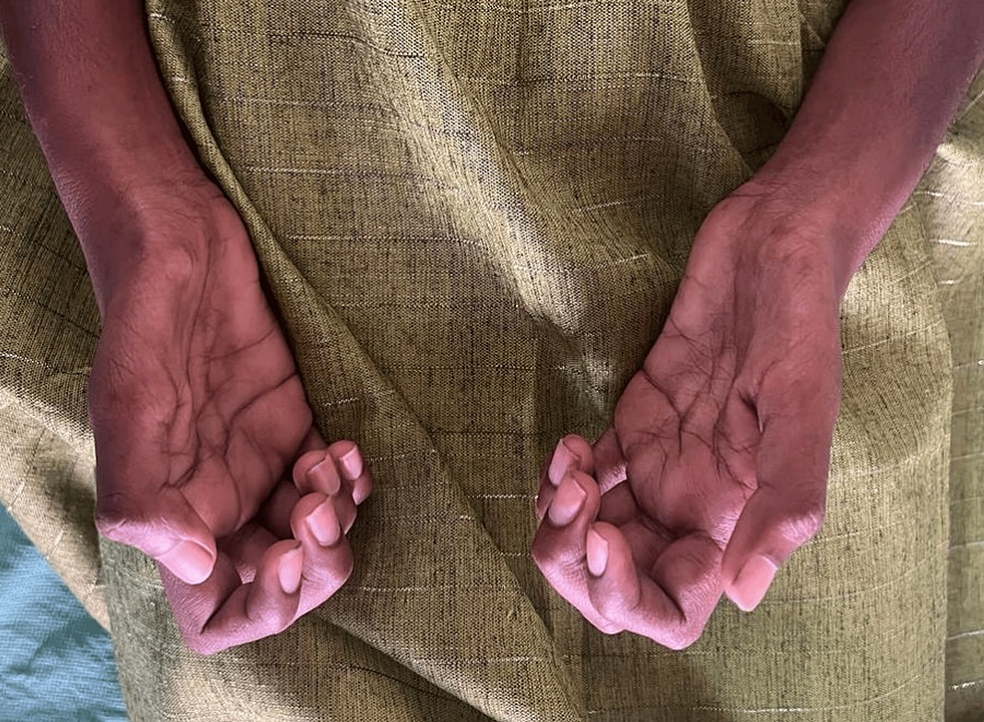
Wasting of thenar and hypothenar muscle

A female patient, 18 years old, arrived at the hospital with a one-year history of increasing muscle atrophy and weakness that was causing her to have trouble walking. The patient, who had previously been in good condition and had no significant medical history, described a gradual development of symptoms, with her upper limbs showing the most atrophy (Figure 1) and weakness at first. Eventually, the weakness spread to her lower extremities, making it difficult for her to walk independently.

Inquiring further, the patient disclosed subjective complaints of bilateral hearing loss, which she had observed at the same time that her motor symptoms started to manifest. It is noteworthy that there was no prior history of trauma, ototoxic drug usage, or loud noise exposure and there was no history of similar complaints in her family.

Upon examination, Higher mental functions were normal, the patient showed signs of weakness and muscle loss in both the upper and lower limbs. A neurological exam showed decreased muscle tone, bulk, and generalized weakness, especially in the distal muscles. Both upper and lower limb muscle power were 3/5, superficial reflexes were normal, and bilaterally exaggerated deep tendon reflexes were seen in both upper and lower limbs, bilateral plantar response was extensor. Fasciculations were seen in the tongue (Video 1) and legs, indicating lower motor neuron involvement.

**Video 1:** Video of the patient suggesting diffuse tongue fasciculations

**Figure 2:**
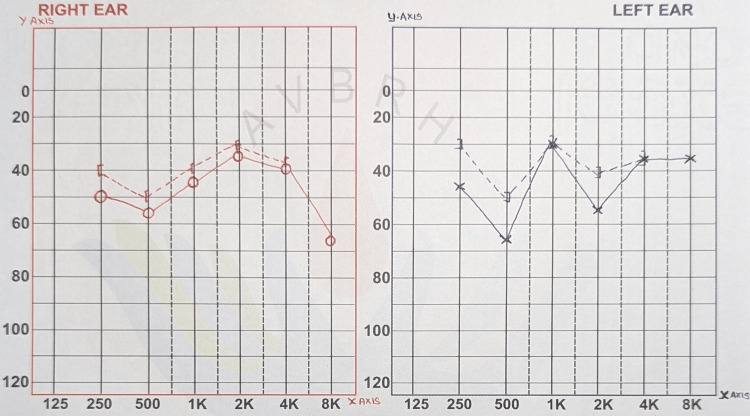
Audiogram of the patient suggestive of moderate sensory neural loss

**Figure 3:**
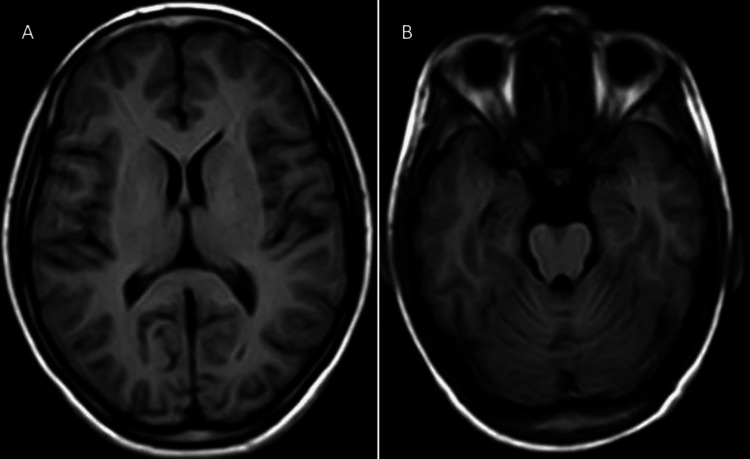
Normal MRI brain of the patient (A, B) MRI: Magnetic Resonance Imaging

**Figure 4:**
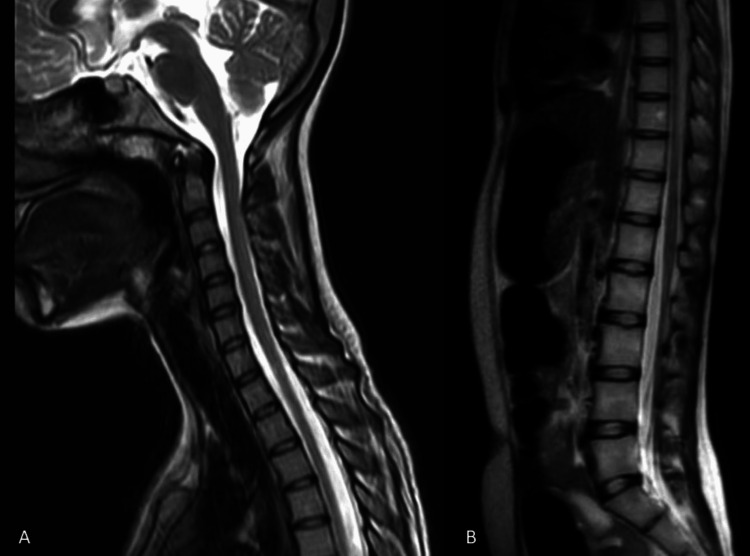
Normal MRI of cervical spine (A) and MRI whole spine screening (B) MRI: Magnetic Resonance Imaging

Given the constellation of symptoms, including progressive muscle weakness, wasting, and bilateral sensorineural hearing loss, a comprehensive diagnostic workup was initiated to elucidate the underlying etiology. All the blood investigations were within normal limits (Table 1). Nerve conduction studies (NCS) were performed. Suggestive motor axonal polyneuropathy (Table 2) and Electromyography (EMG) were performed and suggestive of anterior horn cell disease affecting three segments (bulbar, cervical, lumbosacral) (Table 3), Audiometry confirmed bilateral sensorineural hearing loss (Figure 2), predominantly affecting high-frequency sounds, with no evidence of conductive hearing impairment. Magnetic resonance imaging (MRI) of the brain and spinal cord was undertaken to rule out alternative diagnoses and assess for structural abnormalities which came out to be normal (Figures 3A, 3B, 4A, 4B). The patient was put on supportive treatments, such as limb physiotherapy and hearing aids because there is no known cure for MMND.

**Table 1:** Investigation profile of the patient

Investigations	Patient	Reference values
Hemoglobin	11.6 g/dL	13-15 g/dL
Total leukocyte count	7,400/dL	4,000-11,000/dL
Platelet count	158,000/dL	150,000-400,000/dL
Serum creatinine	0.5 mg/dL	0.5-1.2 mg/dL
Albumin	2.9 g/dL	3.5-5.0 g/dL
Aspartate Aminotransferase	28 U/L	<50 U/L
Alanine Aminotransferase	21 U/L	17-59 U/L
Total Bilirubin	0.4 mg/dL	0.2-1.3 mg/dL
C Reactive Protein	4mg/dL	<6 mg/dL
Erythrocyte Sedimentation Rate	1 mm/hr	0-2 mm/hr

**Table 2:** Electromyography summary table of the patient IA: Insertion Activity, Fib: Fibrillations, PSW: Polyspike Wave, Fasc: Fasciculations,H.F.: High Frequency, Amp: Amplitude, Dur: Duration, PPP: Polyphasic Potential, R: Right, L: Left, N: Normal

EMG summary table			Spontaneous					MUAP			Recruitment
Muscle	Nerve	Roots	IA	Fib	PSW	Fasc	H.F.	Amp	Dur.	PPP	Pattern
R. Tibialis anterior	Deep peroneal (Fibular)	L4-L5	N	None	None	None	None	N	N	N	N
L. Quadriceps	Femoral	L2-L4	N	None	None	None	None	N	N	N	Reduced
R. Biceps brachii	Musculocutaneous	C5-C6	N	None	None	None	None	N	N	N	N
R. First dorsal interosseous	Ulnar	C8-T1	N	None	None	None	None	N	N	N	N
R. Tongue	Hypoglossal	Medulla	N	2+	None	None	None	N	N	N	N
L. Abductor pollicis brevis	Median	C8-T1	N	3+	None	None	None	N	N	N	N

**Table 3:** Motor nerve conduction study of the patient APB: Abductor pollicis brevis, ADM: Abductor digiti minimi, EDB: Extensor digitorum brevis, AH: Abductor hallucis, NR: Not recordable

Nerve/sites	Muscle	Latency (ms)	Amplitude (mV)	Segments	Dist. (mm)	Lat Diff (ms)	Velocity (m/s)
Right Median - APB							
Wrist	APB	4.71	0.3	Wrist - APB	80		
Elbow	APB	NR	NR	Elbow - Wrist		NR	
Left Median - APB							
Wrist	APB	3.10	1.3	Wrist - APB	80		
Elbow	APB	NR	NR	Elbow - Wrist		NR	
Right Ulnar - ADM							
Wrist	ADM	2.48	2.1	Wrist - ADM	80		
B. Elbow	ADM	6.23	2.1	B. Elbow - Wrist	220	3.75	58.7
Left Ulnar - ADM							
Wrist	ADM	2.75	1.7	Wrist - ADM	80		
B. Elbow	ADM	6.92	1.5	B. Elbow - Wrist	220	4.17	52.8
Right Peroneal - EDB							
Ankle	EDB	4.04	2.0	Ankle - EDB	80		
B. Fib Head	EDB	10.35	2.0	B. Fib Head - Ankle	310	6.31	49.1
Left Peroneal - EDB							
Ankle	EDB	3.96	1.8	Ankle - EDB	80		
B. Fib Head	EDB	9.19	1.1	B. Fib Head - Ankle	310	5.23	59.3
Right Tibial - AH							
Ankle	AH	2.81	10.1	Ankle - AH	80		
Knee	AH	10.98	8.2	Knee - Ankle	340	8.17	41.6
Left Tibial - AH							
Ankle	AH	4.13	10.6	Ankle - AH	80		
Knee	AH	11.04	9.7	Knee - Ankle	340	6.92	49.2

## Discussion

MMND, which makes up 0.9%-3.7% of all MND patients, is a unique kind of disease [3]. MMND is frequently characterized by early onset, unpredictable incidence, sensorineural hearing loss, multiple lower cranial nerve palsy, generalized atrophy with limb weakness, and a benign but progressive history. While MMND and ALS have certain characteristics, they differ in terms of age at onset, facial palsy, and deafness. Our patient did not initially exhibit ocular atrophy; however, some cases do have cerebellar involvement.

A small number of MMND patients have been reported from Pakistan (Khan and Rashid, 2013), Thailand (Phanthumchinda et al., 1996), Italy (Massa et al., 1998), China (Fan et al., 2004; He et al., 2015), Korea (Im and Kim, 2008), Turkey (Isak et al., 2009), and Pakistan (Fan et al., 2004) [3-5]. Notably, the majority of MMND cases are centered in southern India, giving rise to a distinctive geographic distribution for the disease. The illness's pathophysiology is still unknown. Since familial MMND (FMMND) has also been recorded, genetic alterations should be taken into consideration even if the majority of reported instances seem to be sporadic (Massa et al., 1998) and 13.8% of cases (including non-familial and familial MMND) show parent consanguinity (Nalini et al., 2008) [3].

Furthermore, Valmikinathan et al. proposed that altered citrate metabolism may be linked to the pathogenesis of MMND [6]. A few case reports speculate that MMND may have an inflammatory etiology. There is a chance that an unidentified C9ORF72 expanded repeat is involved [2]. Nonetheless, a mix of environmental and genetic elements is believed to have causal effects. There is no proven cure for this type of MND, like for others. When compared to ALS, MMND progresses more slowly and has a comparatively longer survival duration. However, a few patients did have a brief survival time. Compared to men, women have a shorter survival duration.

The clinical characteristics of this patient are consistent with MMND manifestations that have been previously documented, such as fasciculations, lower motor neuron signs, and increasing muscle weakness and wasting. Notably, tongue fasciculations complicate the diagnosis since, while it can occur in other neurological disorders, it is frequently linked to MND. Our patient's concurrent bilateral sensorineural hearing loss broadens the clinical spectrum of MMND and emphasizes the possibility of multisystem involvement in addition to motor neurons [7].

Due to the lack of disease-modifying therapies at this time, managing MMND presents substantial hurdles. To enhance quality of life and reduce symptoms, supportive care and symptom management are the main goals of therapeutic interventions. Physical therapy, mobility assistive devices, and communication aids are some interventions that could help our patient maximize their independence and minimize their impairment [8].

Brown-Vialetto-Van Laere (BVVL) syndrome, Boltshauser syndrome, Nathalie syndrome, and Fazio-Londe syndrome are common anterior horn cell diseases mimicking MMND [2]. Clinically, BVVL syndrome is similar to MMND. However, it is caused by mutations in SLC52A2 (encoding RFVT2) or SLC52A3 (encoding RFVT3), which results in a riboflavin transporter defect that improves with riboflavin therapy, halting the progression of this uncommon neurodegenerative illness [2].

To better understand the natural history of MMND and find potential prognostic markers, more studies are necessary as prognostic variables and illness progression are still poorly understood, especially in younger patients. Moreover, it emphasizes the necessity of sustained efforts to broaden our awareness of MMND pathophysiology, formulate specific therapeutics, and enhance the prognosis for affected persons.

## Conclusions

The clinical variability and diagnostic difficulties related to this uncommon MND are brought to light by the MMND presentation in an 18-year-old patient who also had concomitant sensorineural hearing loss. Clinicians should be reminded by this example to keep an open mind when diagnosing patients, especially young people who appear with progressive motor and sensory deficits, and to exclude MMND. To improve our knowledge of MMND and create efficient treatment plans for this disabling illness, more research is necessary.
